# Explainable multimodal AI and neuro-symbolic clinical decision support system for chronic eye disease management: a digital health implementation study

**DOI:** 10.3389/fdgth.2026.1836890

**Published:** 2026-07-09

**Authors:** Mini Han Wang, Simon Ming Yuen Lee, Guanghui Hou, Yapeng Wang, José C. Alves, Ruitao Xie, Yaqing He, Jin Liu, Xiaoxiao Fang, Yu Yang, Xiaodong Cai, Shuai Zheng, Ziyang Yu, Ethan Zhiyuan Lin, Chonin Cheang, Kuok Kai Ian, Shuai Qin

**Affiliations:** 1 The Hong Kong Polytechnic University, Hong Kong, Hong Kong SAR, China; 2 Zhuhai Institute of Advanced Technology Chinese Academy of Sciences, Zhuhai, Guangdong, China; 3 Zhuhai People’s Hospital (The Affiliated Hospital of Beijing Institute of Technology, Zhuhai Clinical Medical College of Jinan University), Zhuhai, Guangdong, China; 4 Shenzhen University of Advanced Technology, Shenzhen, China; 5 Aier Eye Hospital of Zhuhai, Zhuhai, Guangdong, China; 6 Faculty of Applied Sciences, Macao Polytechnic University, Macau, Macau SAR, China; 7 Faculty of Business, City University of Macau, Macau, Macau SAR, China; 8 Faculty of Computer Science and Artificial Intelligence, Shenzhen University of Advanced Technology, Shenzhen, China; 9 Hong Kong Productivity Council, Smart City Division, Hong Kong, Hong Kong SAR, China; 10 Hunan Provincial Key Lab on Bioinformatics, School of Computer Science and Engineering, Central South University, Changsha, China; 11 Department of Orthopedic Spinal Surgery, Nanfang Hospital, Southern Medical University, Guangzhou, China; 12 School of Integrated Circuits and Electronics, Beijing Institute of Technology, Beijing, China; 13 OmniContext AI Lab, Hong Kong, Hong Kong SAR, China; 14 Macau Society for Health Economics, Macau Yinkui Hospital, Macau, Macau SAR, China; 15 Macau Yinkui Hospital, Macau, Macau SAR, China; 16 Xiaoao Technology Co., Ltd., Macau, Macau SAR, China; 17 Department of Ophthalmology, The Third Affiliated Hospital of Southern Medical University, Guangzhou, China

**Keywords:** administrative automation, age-related macular degeneration, clinical documentation, digital health, explainable AI, health informatics, large language models, neuro-symbolic AI

## Abstract

**Introduction:**

Administrative burden and documentation workload are increasingly recognized as major contributors to healthcare costs and clinician burnout, particularly in chronic disease management such as age-related macular degeneration (AMD). These non-clinical tasks reduce time available for patient care and introduce inefficiencies in coding, billing, and compliance workflows. This study aimed to evaluate the technical feasibility and economic impact of an artificial intelligence–based system for automating administrative documentation in AMD care.

**Methods:**

A longitudinal proof-of-concept study was conducted in a tertiary ophthalmology network. A hybrid Neuro-Symbolic and Large Language Model (LLM) framework was developed to automate information extraction, clinical documentation structuring, and billing code validation. The system was applied to 24 de-identified unstructured clinical documents, including outpatient notes, operative reports, optical coherence tomography reports, and billing records. The processing pipeline included optical character recognition, knowledge-augmented LLM-based entity extraction, and neuro-symbolic rule-based validation to ensure clinical consistency, ICD-10/CPT coding accuracy, and reimbursement eligibility. Outputs were structured into standardized JSON clinical documentation formats.

**Results:**

The system achieved 98.3% accuracy in clinical entity extraction and 96.7% accuracy in administrative information extraction. Automated rule validation achieved 100% reimbursement compliance with no denied insurance claims. Mean documentation time per encounter decreased from 25.0 ± 5.0 min to 3.2 ± 1.1 min, representing an 88% reduction in documentation time. This resulted in an estimated labor cost saving of approximately 52 CNY (≈7 USD) per visit and projected annual savings of 42–56 USD per AMD patient under standard treatment schedules.

**Discussion:**

This study demonstrates the feasibility of integrating neuro-symbolic reasoning with large language models to automate administrative workflows in ophthalmology. The proposed system improves documentation efficiency, coding accuracy, and auditability while reducing hidden administrative costs. Although limited by a small sample size and single-center design, the framework provides a scalable and explainable architecture for AI-assisted administrative automation in digital health systems and chronic disease management.

**Conclusion:**

AI-enabled administrative automation using a neuro-symbolic and LLM framework has the potential to significantly improve operational efficiency and reduce costs in AMD care, supporting the development of sustainable and value-based digital healthcare systems.

## 1 Introduction

The strategic application of AI and Machine Learning presented in this study is vital for transforming the management of chronic conditions as it directly augments healthcare capacity by freeing clinicians from bureaucratic burdens, which in turn reduces treatment delays and prevents vision loss. Age-related macular degeneration (AMD) (1) represents one of the most prevalent causes of irreversible blindness (2) in the ageing population and constitutes a rapidly escalating public-health (3) and economic challenge. Epidemiological modelling projects that the number of individuals affected by AMD (1) will rise to more than 280 million by 2040 (4), driven by demographic ageing and lifestyle-related risk factors (5). The economic burden of AMD is correspondingly immense (1). When the costs of direct ophthalmic care, long-term pharmacotherapy (6), visual rehabilitation, informal caregiving, and loss of work productivity (7) are aggregated, global expenditures are expected to reach tens of billions of U.S. dollars annually. Current standards of care (8)—principally repeated intravitreal injections of anti-vascular endothelial growth factor (anti-VEGF) (9) agents combined with high-frequency optical coherence tomography (OCT) imaging (10)—are clinically effective but create substantial, recurring costs for both public health systems and private payers (11).

While clinical treatment constitutes the most visible expenditure (12), a less recognized yet critical economic driver is the administrative workload (13) associated with chronic AMD management (14). Each injection cycle requires meticulous documentation of diagnostic findings, justification of therapeutic decisions, and detailed reporting of follow-up outcomes to satisfy reimbursement policies, pay-for-performance programmes, and medico-legal auditing (15). Manual preparation of operative notes, cross-temporal comparison of imaging biomarkers, and submission of billing or insurance claims are labour-intensive and error-prone processes. Because patients frequently undergo multiple injections over many years, these tasks scale geometrically, consuming clinician and technician time, delaying claims processing, and contributing significantly to hidden transaction costs (16) that erode the overall efficiency of eye-care delivery.

Advances in hybrid artificial intelligence (17) provide a compelling pathway (18) to address this structural inefficiency (19). Neuro-symbolic AI (20) integrates the explicit logical reasoning of symbolic systems (21) with the adaptive pattern-recognition capabilities of deep neural networks (22), enabling machines to represent and manipulate domain knowledge (23) while learning from heterogeneous clinical data (24). Large language models (LLMs) (25) complement this framework by understanding and generating complex free-text narratives, extracting key clinical indicators (20), and producing coherent, human-readable documentation (26). When combined, these technologies can automatically convert unstructured ophthalmic records into structured, machine-verifiable data (27); apply codified reimbursement and quality-assurance rules; and generate explainable (28) audit-ready reports (29). Such an integrated neuro-symbolic–LLM (30) architecture promises not only to alleviate manual workload (31) and reduce direct administrative costs, but also to improve traceability (32), reproducibility (33) and regulatory compliance (34) across the continuum of AMD care. By embedding transparent reasoning (20) and economic accountability into routine documentation, this approach aligns with the strategic aims of modern health-economics policy (35): to deliver administratively efficient (36), high-quality (37), and scalable eye-care services for an ageing global population.

**Figure 1:**
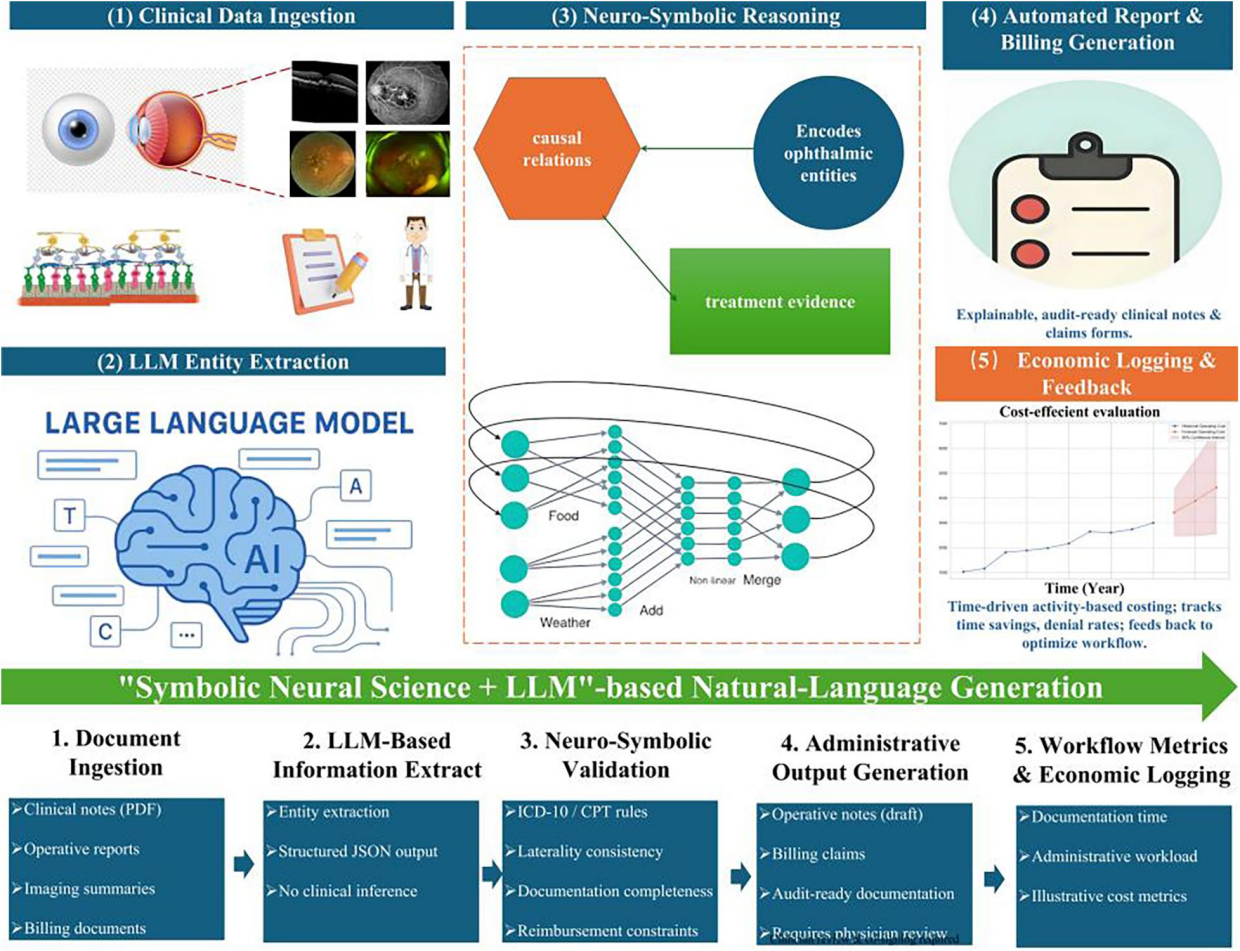
Administrative automation pipeline using a neuro-symbolic+LLM framework.

This study presents a technical feasibility evaluation of a hybrid Neuro-Symbolic and Large Language Model framework designed to automate healthcare administrative workflows (Figure 1) in age-related macular degeneration care. The proposed system focuses exclusively on documentation processing, coding validation, and reimbursement-related tasks, without engaging in diagnostic reasoning or treatment decision-making. By integrating LLM-based information extraction with explicit neuro-symbolic rule validation, the framework enables transparent, auditable transformation of unstructured clinical records into structured, reimbursement-compliant administrative outputs. Within a single-case longitudinal setting, the study demonstrates descriptive, within-case improvements in documentation completeness, administrative workload, and workflow traceability, while explicitly avoiding claims of population-level effectiveness or economic generalizability. Collectively, this work highlights the potential role of explainable, policy-aligned AI architectures in reducing administrative burden surrounding clinical care.

Figure 1 illustrates the end-to-end workflow for processing unstructured clinical and billing documents into structured, audit-ready administrative outputs. The pipeline supports documentation completeness, coding validation, and reimbursement compliance. It is designed to assist administrative workflows and does not represent diagnostic reasoning or treatment decision-making.

This paper is organized as follows. Section 2 describes the materials and methods, including the single-case study design, data sources, reference standard and validation process, neuro-symbolic knowledge representation, LLM integration, end-to-end workflow, implementation details, economic perspective, and predefined feasibility outcomes. Section 3 reports the results of the feasibility evaluation, presenting descriptive, within-case findings related to information extraction, rule-based compliance, documentation workload, and illustrative administrative cost implications. Section 4 discusses the implications of these findings in the context of administrative automation, explainability, and health-system governance, and articulates the rationale for the Neuro-Symbolic+LLM architecture. Section 5 outlines the study's limitations and directions for future research, including the need for multi-center validation and comparative evaluation. Finally, the conclusion summarizes the key feasibility insights and situates the work within broader efforts to responsibly deploy AI for healthcare administration.

This work introduces several novel architectural patterns for AI-enabled administrative automation in healthcare. First, we propose a constraint-gated extraction loop, in which LLM–based information extraction is tightly coupled with a neuro-symbolic validation layer that deterministically accepts, flags, or rejects field-level outputs based on predefined logical constraints, thereby enhancing auditability and reducing unsafe free-form variability. Second, we present a field-typed structured decoding strategy, combining prompt design and post-processing to enforce schema-typed outputs—such as explicit field types, controlled enumerations, and laterality attributes—minimizing downstream ambiguity in administrative workflows. Third, we elevate administrative compliance to a first-class reasoning target, explicitly encoding ICD-10/CPT compatibility and reimbursement eligibility rules at the encounter level, rather than treating coding as a *post hoc* or purely statistical task. Together, these contributions extend existing neuro-symbolic and LLM-based approaches by aligning AI reasoning with the practical constraints of real-world clinical administration.

## 2 Materials and methods

### 2.1 Study design and setting

This investigation was conceived as a single-case, longitudinal study designed to showcase the practical application of a hybrid Neuro-Symbolic and LLM framework for automating administrative tasks in the management of AMD. The approach allows detailed monitoring of clinical events over time, enabling the capture of dynamic changes in both medical outcomes and administrative processes. By focusing on a single, richly documented case, the study provides a high-resolution proof of concept for integrating advanced artificial-intelligence tools into routine ophthalmic care while laying the foundation for future multi-center and large-scale evaluations.

The research was conducted within a tertiary ophthalmology network affiliated with Zhuhai People’s Hospital, a regional referral center equipped for advanced retinal diagnostics and therapy. The clinical context involved Subject 1 (fully de-identified to protect privacy), who was diagnosed with AMD complicated by recurrent macular edema and treated with three intravitreal injections of anti-VEGF agents over a three-month observation period. Each treatment cycle included comprehensive preoperative assessment, intraoperative documentation, and postoperative follow-up, generating a complete longitudinal record of the disease course and associated administrative workflow.

All clinical information—including outpatient encounter notes, operative reports, imaging interpretations (OCT/OCTA), medication logs, and insurance billing statements—was originally stored as unstructured PDF files within the hospital's electronic health record and administrative systems. These heterogeneous and largely narrative documents typify the data sources encountered in everyday ophthalmic practice and therefore provide an authentic substrate for testing automated extraction and reasoning methods.

The OCR pipeline was implemented using PaddleOCR v2.7, while the LTN reasoning layer was developed using TensorFlow 2.13 and Keras 2.13. The knowledge graph contained 218 nodes, 545 edges, and 12 relation types. The final symbolic validation layer incorporated 37 logical constraints and administrative validation rules.

Formal ethical clearance for the study protocol was obtained from the Ethics Committee of Zhuhai People’s Hospital [approval number: (2024)-KT-67]. The committee reviewed all methodological details, including data de-identification procedures and the intended secondary use of clinical records, and confirmed that the study complied with the Declaration of Helsinki and relevant Chinese regulations on medical research. The patient's identity was concealed throughout the study by replacing all personal identifiers with the code Subject 1, and data were stored on secure, access-controlled servers to safeguard confidentiality.

By combining a carefully documented clinical course with rigorous ethical oversight and secure data handling, this single-case longitudinal design provides a robust and transparent platform to evaluate how neuro-symbolic reasoning coupled with LLM-based language understanding can transform administrative processes in AMD care, reduce hidden costs, and improve overall efficiency within a real-world tertiary care setting. Structured outputs followed a predefined JSON schema describing clinical entities, administrative codes, validation flags, and timing/economic variables (Supplementary File S1). An example de-identified JSON instance illustrating the structured outputs for representative encounters is provided in Supplementary File S2.

### 2.2 Data sources and preprocessing

All clinical and administrative records generated throughout the entire course of care—from the initial diagnosis through three intravitreal anti-VEGF injections and the intervening follow-up visits—were systematically exported in portable document format (PDF) from the hospital's electronic medical record and billing systems. The exported dataset encompassed outpatient consultation notes, operative and anesthesia reports, OCT and other imaging summaries, postoperative progress notes, and itemized insurance or reimbursement statements. By capturing each encounter as it occurred, this comprehensive corpus provided a temporally ordered account of both the clinical trajectory and the associated administrative workload.

Prior to analysis, rigorous de-identification procedures were applied to ensure compliance with institutional and international privacy standards. All personally identifiable information—including the patient's name, medical record number, date of birth, contact information, and any free-text references that could indirectly reveal identity—was removed or replaced with the neutral code Subject 1. The de-identification process was independently verified by the data management team and documented in accordance with the requirements of the Zhuhai People's Hospital Ethics Committee [approval number (2024)-KT-67].

Because many of the records contained mixed formats such as scanned handwritten notes or embedded image-based diagrams, optical character recognition (OCR) was applied when necessary to convert non-searchable PDFs into machine-readable text. The OCR step enabled downstream computational parsing and ensured that no clinically relevant information was excluded due to format constraints. All resulting text outputs were manually spot-checked against the original files to confirm accuracy of conversion, particularly for numeric values such as visual acuity measurements, intraocular pressure readings, and drug dosages.

The curated text corpus was then indexed chronologically to reflect the patient's clinical pathway from December 2024 through March 2025. Each record was annotated with visit type (e.g., preoperative, intraoperative, postoperative, or follow-up) and systematically linked to key health-economic descriptors, including staff time devoted to documentation, estimated labor costs, reimbursement amounts, and claim approval or rejection dates. This structured linkage between clinical content and economic metrics allowed for precise cost analysis, time-driven activity-based costing, and evaluation of administrative efficiency. Collectively, these preprocessing steps created a high-quality, privacy-compliant dataset ideally suited for subsequent Neuro-Symbolic reasoning and LLM–based automation as well as for robust health-economic assessment.

### 2.3 Reference standard and validation

To support a reproducible and feasibility-oriented evaluation, a reference standard was constructed to assess the correctness of the system's structured outputs at the field level. The reference standard consisted of encounter-level JSON records aligned with the predefined schema used by the Neuro-Symbolic and LLM pipeline, enabling direct, structured comparison between automated outputs and expert annotations.

Reference annotation was performed by two independent ophthalmologists from different clinical institutions to reduce institutional bias. One annotator was an ophthalmologist from Zhuhai Aier Eye Hospital, and the second was an ophthalmologist from Zhuhai People’s Hospital. Both annotators independently reviewed the same set of de-identified clinical and administrative documents and produced structured annotations following a shared annotation guideline. The guideline specified field definitions, normalization rules, and acceptable value formats, including visual acuity normalization (e.g., ETDRS letter conversion), unit handling for OCT-derived measurements, and standardized encoding of laterality and procedural attributes.

The annotated variables included both clinical entity fields and administrative documentation fields relevant to AMD care. Clinical entities encompassed best-corrected visual acuity, intraocular pressure, OCT biomarkers such as central macular thickness and the presence or absence of intraretinal and subretinal fluid, as well as treatment-related attributes including anti-VEGF agent, injection date, and laterality. Administrative fields included diagnosis coding information (ICD-10), procedure-related descriptors, encounter dates, laterality indicators, and documentation completeness elements required for reimbursement submission, such as mandatory operative-note components and medication traceability fields when available in the source records.

Agreement between automated outputs and the reference standard was evaluated at the field level using exact-match criteria after normalization. Continuous variables were considered matching when extracted values and units were consistent with the reference standard after applying predefined conversion and formatting rules. Categorical variables, including laterality indicators and coding fields, were evaluated as exact matches. Results are reported as raw counts (X/Y) and corresponding descriptive agreement percentages, consistent with the engineering-oriented feasibility framing of this study. Inter-annotator agreement was assessed on the labeled reference standard using Fleiss' *κ*, reflecting agreement among three independent annotators. Agreement was evaluated separately for clinical entity labels and administrative descriptor labels. The resulting agreement coefficients were κ=K1(95%CI:CI1–CI2) for clinical entities and κ=K2(95%CI:CI3–CI4) for administrative descriptors, indicating substantial agreement across annotators. In cases of disagreement, final labels were determined through consensus adjudication by a senior domain expert, ensuring the reliability of the reference standard used for downstream accuracy evaluation.

Given the single-case design, a stratified sampling strategy was applied for manual validation to ensure representative coverage across document types and encounter stages while maintaining feasibility. Specifically, 20% of documents were selected using stratification by encounter type (initial diagnosis, intravitreal injection visits, and follow-up visits) and by document category (clinical notes, imaging summaries, operative reports, and billing-related documents). This stratified subset was used for detailed manual comparison, while all documents were additionally subjected to rule-based validation through the neuro-symbolic reasoning layer.

Inter-annotator agreement was assessed on the overlapping annotated subset prior to adjudication using simple percentage agreement at the field level, separately for clinical entity fields and administrative documentation fields. Discrepancies between annotators were resolved through joint review and adjudication to produce a single reconciled reference JSON per encounter. Most disagreements arose from ambiguities or omissions in the original clinical documentation rather than from systematic differences in clinical interpretation. The adjudicated reference standard served as the comparator for all reported within-case descriptive agreement results.

### 2.4 Neuro-Symbolic knowledge representation

To enable explainable, rule-driven automation of administrative tasks in chronic retinal disease management, we developed a domain-specific neuro-symbolic knowledge representation framework tailored to age-related macular degeneration (AMD) care. The foundation of this framework was an ophthalmology-specific knowledge graph (KG) that formalizes the relationships among disease entities, imaging biomarkers, therapeutic procedures, documentation artifacts, and reimbursement requirements relevant to longitudinal AMD management.

The KG encoded standardized diagnostic entities, including neovascular AMD, recurrent macular edema, and retinal vascular disorders associated with anti-vascular endothelial growth factor (anti-VEGF) treatment workflows. Diagnosis and procedure entities were explicitly anchored to established coding systems, including the International Classification of Diseases, Tenth Revision (ICD-10), and Current Procedural Terminology (CPT) identifiers, thereby facilitating interoperability with electronic medical record systems and insurance billing workflows.

In addition to diagnoses and procedures, the KG incorporated clinically relevant imaging biomarkers derived from optical coherence tomography (OCT), including central macular thickness (CMT), intraretinal fluid (IRF), subretinal fluid (SRF), and other treatment-monitoring features. For each biomarker, the KG specified associated measurement units, clinically meaningful thresholds, and longitudinal interpretation states such as improvement, stability, or progression. Treatment events, including intravitreal anti-VEGF injections and ancillary retinal procedures, were represented as temporally linked entities with attributes describing medication type, injection date, procedural laterality, and treatment sequence.

Unlike broad biomedical ontologies that prioritize exhaustive semantic coverage, the proposed KG adopted a task-oriented operational scope optimized for AMD administrative automation. The symbolic schema was intentionally designed according to an operational sufficiency principle, whereby only entities and relations required for downstream validation, coding consistency, reimbursement eligibility assessment, and longitudinal workflow reasoning were explicitly formalized. Candidate symbols, predicates, and semantic relations were initially identified from publicly available ophthalmology guidelines, ICD-10/CPT coding manuals, OCT reporting templates, institutional documentation protocols, and anti-VEGF reimbursement policies. These entities and constraints were subsequently reviewed and refined by retinal specialists and clinical informatics researchers to ensure consistency with real-world AMD documentation workflows.

The final symbolic schema encompassed the core structured fields extracted by the large language model (LLM), including diagnoses, procedures, medication information, OCT biomarkers, laterality attributes, encounter dates, billing codes, and documentation completeness indicators. Rather than attempting exhaustive biomedical representation, the symbolic layer focused specifically on clinically and administratively actionable entities relevant to automated validation and reimbursement reasoning. Extracted entities that could not be mapped to predefined predicates or logical constraints were conservatively flagged for human review rather than being automatically validated, thereby reducing the risk of unsupported deterministic decisions.

To transform these structured relationships into a machine-reasoning layer, administrative validation rules and workflow constraints were formalized using first-order logic and implemented within a Logical Tensor Network (LTN) framework. Candidate logical rules were derived from institutional billing workflows, ICD-10/CPT coding standards, reimbursement eligibility policies, and ophthalmology documentation requirements. These rules were subsequently reviewed and refined through retrospective encounter simulation and expert evaluation. The final rule set focused on high-frequency administrative validation scenarios, including diagnosis–procedure compatibility, laterality consistency, mandatory OCT evidence prior to injection reimbursement, allowable retreatment intervals, operative note completeness, and billing eligibility verification.

Representative examples of the logical constraints used in the neuro-symbolic validation framework are summarized in Table 1, while additional rule definitions are provided in Supplementary Table S1.

**Table 1:** Representative first-order logical constraints used in the neuro-symbolic validation framework.

Rule ID	Description	First-Order Logic
R1	Injection requires valid diagnosis	Proc(e,Injection) → ∃d Dx(e,d)
R2	Laterality consistency	DxLat(e,l) ∧ ProcLat(e,l)
R3	OCT required before reimbursement	Claim(e) → OCT(e)
R4	Re-treatment interval constraint	Interval(e₁,e₂) ≥ 28 days
R5	CPT requires operative note	CPT(e,c) → NoteComplete(e)

Although many administrative constraints could theoretically be implemented using conventional deterministic programming logic, the Logical Tensor Network framework was selected because the upstream clinical entities were extracted from unstructured clinical text using probabilistic LLM-based extraction. In this setting, diagnoses, procedures, laterality attributes, and imaging findings may contain uncertainty, ambiguity, or incomplete confidence. Unlike conventional rule engines that operate using binary true/false logic, LTNs represent predicates as continuous truth values within the interval [0,1], thereby enabling uncertainty-aware symbolic reasoning and differentiable soft constraint evaluation. This neuro-symbolic formulation provides a principled bridge between probabilistic neural extraction and structured administrative validation, allowing symbolic constraints to interact directly with uncertain extraction outputs while preserving interpretability, auditability, and extensibility.

The LTN component was implemented using a TensorFlow/Keras-based framework and functioned primarily as a differentiable symbolic validation layer rather than an independent predictive classifier. Predicate truth values were computed from structured outputs extracted by the LLM pipeline and evaluated against predefined logical constraints. During system development, logical formulations and threshold parameters were iteratively refined through retrospective encounter simulations and expert review to minimize false validation conflicts while preserving rule consistency. Because the present study focused on workflow feasibility rather than large-scale statistical learning, the LTN was used primarily for uncertainty-aware constraint satisfaction and symbolic reasoning rather than end-to-end supervised optimization.

To improve clarity, this study explicitly define the symbols, predicates, and semantics used in the LTN formalization before presenting logical constraints. Clinical encounters are denoted by e, diagnoses by d, procedures by p, and billing codes by c. Predicates such as $D_{x}(e,d)$, Proc(e,p), and CodeValid(c) indicate, respectively, that a diagnosis is asserted in an encounter, a procedure is performed, and a billing code satisfies coding rules. Laterality is represented by Lat(x,ℓ), where ℓ∈{Left,Right,Bilateral,Unspecified}. Under LTN semantics, each predicate evaluates to a continuous truth degree in [0,1], allowing logical constraints to be enforced as soft but explicit validation rules.

As an illustrative example, suppose the LLM extracts the diagnosis $D_{x}(e,H35.32)$ with laterality=Right, while the corresponding injection procedure Proc(e,67028) is assigned laterality=Left. Although the billing code itself may otherwise satisfy coding requirements, the laterality consistency constraint evaluates to a low truth value because diagnosis and procedure laterality do not match. The symbolic validation layer therefore flags the encounter as LATERALITY_INCONSISTENT and rejects reimbursement eligibility until manual correction is performed. When both diagnosis and procedure laterality are consistent, the logical constraint is satisfied and the encounter successfully passes administrative validation. This example illustrates how neuro-symbolic reasoning transforms probabilistic LLM-extracted entities into explicit, auditable, and clinically interpretable administrative decisions.

The resulting AMD task-specific KG contained 218 nodes, 545 edges, and 12 relation types covering diagnoses, imaging biomarkers, medications, procedures, laterality attributes, reimbursement constraints, billing rules, and longitudinal treatment relationships across diagnosis, OCT evaluation, intravitreal injection, follow-up, and claim submission stages. Rare complications, atypical documentation patterns, and off-label treatments were handled conservatively through a human-review fallback mechanism whenever corresponding symbolic constraints were not explicitly encoded within the KG. This design ensured operational safety while maintaining extensibility for future integration of additional biomarkers, payer-specific policies, or ophthalmology subspecialty workflows.

### 2.5 Large language model integration

To complement the neuro-symbolic reasoning layer and to handle the large volume of unstructured clinical narratives, we developed a domain-adapted LLM of DeepSeek-R1 (37) specifically tailored for ophthalmology. The model was based on a state-of-the-art GPT-class transformer architecture and fine-tuned on a bilingual Chinese–English corpus comprising peer-reviewed ophthalmology literature, electronic medical records, surgical reports, and health-insurance guidelines. This bilingual design ensured that the system could seamlessly process documentation in either language and accurately map ophthalmic terminology to international diagnostic and procedural standards.

The LLM was tightly coupled to the ophthalmic knowledge graph through a retrieval-augmented prompting strategy. When a clinical document was ingested, the system first queried the knowledge graph to retrieve relevant disease entities, imaging biomarkers, treatment codes, and reimbursement constraints. These structured elements were dynamically inserted into the model's prompt, guiding text understanding and generation with authoritative, context-specific knowledge. This integration enabled the LLM to perform three critical functions with high accuracy and transparency.

First, the LLM carried out automated extraction of structured variables from heterogeneous free-text sources, such as outpatient notes, operative records, and OCT imaging descriptions. Key variables included visual acuity (both Snellen and ETDRS equivalents), intraocular pressure, central macular thickness, and qualitative descriptors of intraretinal and subretinal fluid. By mapping extracted data to the predefined JSON schema aligned with the knowledge graph, the system ensured consistency, machine readability, and traceability of all clinical measurements.

Second, the LLM was capable of automatic drafting of operative notes and longitudinal comparisons. Leveraging its deep natural-language understanding and the chronological structure provided by the knowledge graph, the model generated detailed, coherent summaries of surgical procedures, postoperative progress, and temporal changes in disease activity. These drafts were designed for clinician verification and could be inserted directly into electronic medical records, thereby reducing manual documentation time while preserving professional standards of accuracy and completeness.

Third, the model enabled the generation of billing codes and accompanying justifications fully consistent with payer-specific rules and international standards such as ICD-10 and CPT. By cross-referencing extracted clinical findings with reimbursement criteria encoded in the knowledge graph, the LLM produced auditable billing statements and claim justifications that satisfied both clinical and administrative requirements. This function not only reduced the likelihood of claim denials but also provided transparent evidence trails for insurance audits and quality-control processes.

Through these synergistic capabilities, the domain-adapted, knowledge-augmented LLM transformed unstructured ophthalmic data into structured, economically actionable information. Its integration with the neuro-symbolic reasoning layer ensured that every output—whether a data table, operative note, or billing claim—was both clinically meaningful and economically compliant, thereby streamlining administrative workflows and supporting administratively efficient care in age-related macular degeneration management.

### 2.6 End-to-End workflow

The proposed system implemented a fully integrated, end-to-end pipeline designed to automate the full spectrum of administrative tasks associated with AMD management. For every clinical encounter—including initial assessment, three intravitreal anti-VEGF injections, and each postoperative follow-up—the framework executed a standardized sequence of operations to ensure data integrity, regulatory compliance, and economic traceability.

Step 1: Entity Extraction. At the point of data ingestion, the domain-adapted LLM processed the raw clinical documents, which included outpatient notes, operative reports, imaging summaries, and ancillary records. Guided by the ophthalmic knowledge graph, the LLM identified and extracted key clinical variables—such as visual acuity (expressed in ETDRS letters), intraocular pressure, OCT descriptors like central macular thickness and fluid status, as well as procedure-related details including injection type, dosage, and laterality. These elements were compiled into a structured JSON output conforming to a predefined schema that supports machine readability and downstream analytics.

Step 2: Rule-Based Validation. The structured data were then subjected to neuro-symbolic rule checking using first-order logic constraints encoded in the Logic Tensor Network. This module validated temporal and logical relationships (e.g., verifying that injection intervals met reimbursement standards), ensured internal consistency across multiple visits, and cross-checked completeness against mandatory documentation requirements. Any discrepancies—such as missing OCT measurements or inconsistent procedure dates—were automatically flagged for clinical review, thereby guaranteeing both clinical soundness and administrative compliance.

Step 3: Automated Report Generation. Following successful validation, the LLM—now constrained by the outputs of the symbolic reasoning engine—generated comprehensive clinical and administrative documentation. This included longitudinal visit summaries, operative notes with embedded evidence citations, and payer-ready billing narratives. The reports were designed to be explainable and audit-ready, providing explicit references to the extracted data and the applied logical rules, which facilitates rapid physician verification and external auditing.

Step 4: Economic Logging. In parallel, the system executed an economic monitoring routine that recorded operational metrics for each encounter. Key parameters included the time required for automated vs. manual documentation, the duration of insurance claim processing, and any claim rejection or appeal events. By systematically linking these data to cost coefficients (e.g., staff wage rates and opportunity costs), the system enabled precise time-driven activity-based costing and facilitated a quantitative evaluation of cost savings and productivity gains attributable to automation.

Through the tight integration of entity extraction, rule-based validation, automated reporting, and economic logging, this end-to-end workflow provides a scalable, transparent, and economically accountable solution for reducing administrative burden in chronic ophthalmic care. It transforms unstructured clinical narratives into actionable, compliant, and cost-analyzable information, demonstrating how neuro-symbolic reasoning and LLM technology can jointly enhance both operational efficiency and health-economic value in AMD management.

### 2.7 Implementation details

The language understanding and generation component of the proposed system was implemented using DeepSeek-R1, a GPT-class transformer-based large language model. The DeepSeek-R1 model (7B parameters) was selected to balance task performance with deployability requirements specific to healthcare administrative workflows. Compared with very large proprietary models, this model size supports on-premise or tightly controlled deployment, enabling compliance with institutional data-governance, auditability, and infrastructure constraints while maintaining low inference latency and operational cost. In addition, the reduced parameter scale facilitates reproducible inference settings and detailed audit logging on commonly available hardware, which is important for administrative applications requiring traceability rather than unconstrained generative flexibility. Inference was performed using a temperature of 0.2 and top-*p* of 1.0, chosen to reduce stochastic variation while preserving sufficient coverage for accurate information extraction from heterogeneous clinical and billing text. Outputs were not assumed to be strictly deterministic. Instead, limited generative variability was controlled through constrained decoding and rendered operationally stable by a deterministic neuro-symbolic validation layer, which enforces schema consistency, logical constraints, and coding rules. Logically inconsistent or non-compliant outputs were automatically rejected or flagged for review, ensuring that clinically and administratively relevant structured fields remained stable across repeated runs, with any residual variability confined to non-critical textual phrasing.

Inference was conducted using conservative and deterministic decoding settings tailored for administrative and regulatory documentation tasks. Specifically, the temperature parameter was set to 0.2, top-p sampling was disabled (top-*p* = 1.0), and the maximum output length was limited to 1,024 tokens per document. These settings were chosen to prioritize consistency, factual stability, and completeness of structured outputs over linguistic variability, which is critical for healthcare administrative applications. Although a non-zero temperature setting (0.2) introduces limited stochasticity in the generation process, output variance was mitigated through constrained decoding, deterministic post-processing enforced by the neuro-symbolic validation layer, and rule-based rejection of logically inconsistent outputs. In practice, repeated executions with identical inputs yielded semantically equivalent structured results, with any residual variability confined to non-critical textual phrasing rather than clinically or administratively relevant fields.

To support domain specificity and reduce hallucination, the LLM was integrated with a retrieval-augmented generation (RAG) mechanism tightly coupled to a domain-specific ophthalmic knowledge graph. For each input document, the system first queried the knowledge graph to retrieve relevant structured context, including candidate AMD disease phenotypes, expected clinical entities and imaging biomarkers, applicable ICD-10 and CPT codes, and reimbursement-related documentation requirements associated with the encounter type. Retrieved knowledge was serialized into a structured prompt prefix that provided authoritative context prior to text parsing and generation. The LLM was instructed to output results in a predefined JSON schema aligned with downstream symbolic validation, ensuring machine readability and interoperability.

All clinical and administrative source documents were provided in portable document format (PDF). For scanned or image-based documents, optical character recognition (OCR) was applied using Tesseract OCR (version 5.3.0) with both English and Simplified Chinese language packs enabled. OCR outputs underwent standardized preprocessing, including page segmentation correction, removal of header and footer artifacts, normalization of numeric formats and units, and correction of common OCR-induced errors. To safeguard data quality, OCR outputs for critical numeric fields—such as visual acuity, intraocular pressure, and OCT-derived measurements—were manually spot-checked prior to downstream processing.

All experiments were executed in a secure on-premise computing environment. Inference and symbolic validation were performed on a workstation equipped with an NVIDIA RTX 3090 GPU (24 GB VRAM), an Intel Xeon CPU, and 128 GB of system memory. The average end-to-end processing time per document, including OCR (when applicable), LLM inference, and neuro-symbolic validation, ranged from approximately 8 to 12 s, depending on document length and structural complexity. Runtime was recorded as part of the feasibility assessment but was not optimized for throughput in this study.

The neuro-symbolic reasoning layer was constructed on top of a domain-specific ophthalmic knowledge graph designed to encode both clinical and administrative knowledge relevant to AMD management. The knowledge graph represented entities such as patients, encounters, disease phenotypes, imaging biomarkers, clinical measurements, procedures, medications, ICD-10 codes, CPT codes, and reimbursement rules. Relationships among these entities encoded clinical associations (e.g., diagnosis-to-procedure), administrative dependencies (e.g., procedure-to-documentation requirements), and reimbursement eligibility constraints. This representation enabled explicit linkage between unstructured clinical findings and structured administrative logic.

Logical reasoning over the knowledge graph was implemented using teh LTN framework, which allowed symbolic rules to be expressed as differentiable first-order logic constraints. These constraints were applied to validate the consistency, completeness, and reimbursement eligibility of structured outputs generated by the LLM. Representative constraints enforced conditions such as allowable intervals between intravitreal anti-VEGF injections for reimbursement eligibility, consistency of eye laterality across clinical notes and billing fields, completeness of documentation elements required by CPT-coded procedures, compatibility between ICD-10 diagnosis codes and AMD phenotype nodes, and the presence of imaging evidence when required for claim justification. For readability, the extracted clinical, administrative, and validation fields are summarized in Table 2, complementing the full JSON schema provided in the Supplementary Materials. An illustrative end-to-end example of the full neuro-symbolic validation workflow, including both a successful pass case and a failed inconsistency case, is presented in Table 3.

**Table 2:** Summary of extracted structured fields.

Field Name	Description	Category
encounter_type	Type of clinical encounter (e.g., initial diagnosis, anti-VEGF injection, follow-up)	Clinical
diagnoses.name	Clinical diagnosis name extracted from documentation	Clinical
diagnoses.icd10	ICD-10 code associated with the diagnosis	Administrative
diagnoses.laterality	Laterality of the diagnosis (left, right, bilateral, unknown)	Clinical
procedures.name	Name of the performed clinical procedure	Clinical
procedures.cpt	CPT/HCPCS code associated with the procedure	Administrative
procedures.laterality	Laterality associated with the procedure	Clinical
medications.name	Medication administered (e.g., anti-VEGF agent)	Clinical
imaging.modality	Imaging modality (e.g., OCT)	Clinical
imaging.key_findings	Key qualitative imaging findings	Clinical
coding.icd10_codes	List of ICD-10 diagnosis codes assigned	Administrative
coding.cpt_codes	List of CPT procedure codes assigned	Administrative
billing.total_charge_amount	Total billed amount for the encounter	Administrative
billing.paid_amount	Amount reimbursed by the payer	Administrative
validation.logical_consistency	Result of neuro-symbolic logical consistency checks	Validation
validation.coding_compliance	ICD-10/CPT coding compliance status	Validation
validation.reimbursement_eligibility	Eligibility of the encounter for reimbursement	Validation
timing.manual_documentation_minutes	Time spent on manual documentation	Validation
timing.automated_minutes	Time required for automated documentation	Validation
economics.per_visit_saving	Estimated labor cost saving per encounter	Validation
audit.physician_review	Physician review and co-signing status	Validation

**Table 3:** Example end-to-end neuro-symbolic administrative validation workflow.

Succuss cases		Failure cases	
Stage	Example	Stage	Example
Original Text	“Right eye intravitreal ranibizumab injection performed.”	Original Text	“Diagnosis: Right eye AMD. Injection performed in left eye.”
LLM Extraction	Diagnosis=H35.32; Procedure=67028; Laterality=Right	LLM Extraction	DxLat=Right; ProcLat=Left
Structured Representation	Dx(e,H35.32), Proc(e,67028), Lat(Right)	Constraint	Laterality consistency
Logical Constraint	∀l DxLat(e,l) → ProcLat(e,l)	LTN Truth Value	0.12
LTN Truth Value	0.98	Validation Outcome	LATERALITY_INCONSISTENT
Validation Outcome	PASS	Billing Decision	Rejected pending correction
Billing Decision	Eligible		

To further support reproducibility, all software dependencies, model checkpoints, and configuration parameters were version-locked, and deterministic inference settings were used throughout the evaluation. No adaptive parameter updates occurred during the study. Together, these implementation details define a transparent and reproducible reference configuration for evaluating AI-enabled administrative automation in ophthalmology.

### 2.8 Cost components and economic perspective

The economic evaluation in this study was conducted from a provider/hospital administrative perspective, focusing specifically on costs associated with documentation, coding, and reimbursement-related workflows surrounding clinical care. This perspective was selected to align with the study's primary objective of assessing the feasibility and administrative impact of AI-enabled workflow automation, rather than evaluating full clinical or societal administratively efficientness.

Cost components included in the analysis comprised clinician and staff time devoted to documentation activities, administrative rework associated with correcting documentation errors, and time spent managing reimbursement processes, including claim submission and resolution of denials. These elements were quantified using a time-driven activity-based costing (TDABC) approach, in which observed task durations were multiplied by local wage-based cost rates to estimate encounter-level administrative labor costs.

Several cost components were explicitly excluded from the analysis. These included system development costs, software integration and customization expenses, staff training, ongoing system maintenance, and computational infrastructure or inference costs associated with running the large language model and neuro-symbolic reasoning engine. These exclusions were made deliberately, as the study was designed as a technical feasibility evaluation rather than a full economic appraisal of system deployment. As such, reported cost values should be interpreted as partial, workflow-level administrative savings rather than net financial benefit estimates.

By clearly delineating included and excluded cost components, this study aims to provide transparent and reproducible estimates of administrative efficiency gains while avoiding overstatement of economic conclusions. A more comprehensive administratively efficientness analysis incorporating development, deployment, and maintenance costs would be required in future large-scale or multi-center implementations.

### 2.9 Feasibility outcomes and evaluation endpoints

In line with feasibility-study reporting norms for health information systems and digital health interventions, this study predefined a set of primary and secondary feasibility outcomes to evaluate the technical readiness, workflow integration, and administrative utility of the proposed Neuro-Symbolic and Large Language Model framework. These outcomes were selected to reflect system-level functionality and administrative impact, rather than clinical effectiveness or patient-level outcomes.

The primary feasibility outcomes focused on the system's ability to operate reliably across heterogeneous documents and encounters. First, successful end-to-end processing rate was defined as the proportion of documents that could be ingested, parsed, structured, validated, and output as compliant JSON records without manual intervention. This metric reflects the technical robustness of the full pipeline, including optical character recognition (when required), LLM-based extraction, and neuro-symbolic validation. Second, documentation completeness and administrative compliance pass rate was defined at the document level as the proportion of processed records meeting predefined ICD-10 and CPT documentation and reimbursement requirements following automated validation and correction. This outcome captures the system's capacity to support accurate and compliant administrative documentation. Third, human review time per encounter was measured as the time required for clinicians to review, verify, and electronically co-sign AI-generated documentation, serving as a direct indicator of administrative workload and workflow efficiency.

Secondary feasibility outcomes were defined to provide additional context regarding system behavior and usability. These included the type and frequency of detected documentation errors, such as ambiguous eye laterality, missing medication lot numbers, incomplete procedural descriptors, or temporal inconsistencies, which were categorized to characterize the nature of administrative issues identifiable by the system. In addition, billing acceptance was recorded as a case-level observational outcome, documenting whether AI-generated claims were accepted on first submission during the study period. Finally, qualitative clinician usability feedback was collected informally from participating ophthalmologists following system use. Clinicians reported that the AI-generated drafts were generally clear, logically structured, and easy to verify, with particular value noted in the consolidation of longitudinal OCT and visual acuity data. Minor feedback related primarily to stylistic preferences and institution-specific documentation conventions, which could be addressed through prompt or template refinement.

To improve transparency and reproducibility, an illustrative end-to-end processing example demonstrating raw clinical text, LLM-based structured extraction, predicate mapping, LTN-based rule evaluation, and administrative validation output is provided in Supplementary Table 3.

Collectively, these feasibility outcomes were designed to assess whether the proposed system could be reliably implemented in a real-world ophthalmology setting, support administrative workflows surrounding clinical care, and generate documentation suitable for clinician review and reimbursement submission. All feasibility outcomes are reported as descriptive, within-case measures and are intended to inform future multi-center evaluations rather than to establish generalizable effectiveness.

### 2.10 Ethical and privacy considerations

This study was conducted in strict accordance with the principles of the Declaration of Helsinki and the institutional guidelines of Zhuhai People's Hospital, ensuring that all procedures involving human data met recognized ethical and legal standards. The complete research protocol, including objectives, data-handling methods, and anticipated dissemination of findings, was reviewed and approved by the Ethics Committee of Zhuhai People's Hospital [approval number: (2024)-KT-67]. This formal approval confirmed that the study satisfied national and international requirements for research involving human participants and the secondary use of clinical records.

To safeguard privacy and confidentiality, all patient-specific identifiers—such as name, address, contact information, and hospital registration number—were removed or replaced with an anonymized code, “Subject 1.” De-identification was performed prior to data import and was verified through automated and manual checks to ensure that no residual personal information could be reconstructed. Only the research team's designated data managers had access to the raw de-identified files, and these files were stored on secure, access-controlled servers with regular audits to detect unauthorized access.

Data processing complied with applicable privacy and data-protection regulations, including relevant Chinese laws and international best practices for handling sensitive health data. All computational analyses, including the neuro-symbolic reasoning and LLM operations, were performed within this secure computing environment. Furthermore, no clinical decision was made solely on the basis of AI output. Every automated report or recommendation generated by the hybrid system was reviewed and verified by qualified ophthalmologists before being used in patient management or administrative submissions. This human-in-the-loop oversight ensured clinical accountability and maintained the primacy of professional judgment in all diagnostic and therapeutic decisions.

By integrating formal ethical approval, rigorous de-identification, secure data stewardship, and mandatory physician verification, the study established a comprehensive ethical and privacy framework. This framework not only protects individual patient rights but also provides a reproducible model for the responsible application of artificial intelligence in healthcare research and practice.

### 2.11 Evaluation protocol (feasibility study)

To ensure appropriate interpretation of quantitative findings within a single-case design, this study adopted an engineering-oriented feasibility evaluation protocol rather than a clinical effectiveness or statistical performance study. The primary objective was to determine whether the proposed Neuro-Symbolic and Large Language Model (LLM) framework could reliably process heterogeneous ophthalmic records, generate structured administrative outputs, and satisfy rule-based validation requirements within a real-world AMD workflow environment. Accordingly, all reported quantitative results should be interpreted as descriptive feasibility observations intended to characterize system functionality, workflow integration, and implementation readiness rather than population-level performance or statistically generalizable accuracy estimates.

The feasibility evaluation was conducted using longitudinal records from a single de-identified AMD patient (Subject 1) who underwent repeated retinal imaging, anti-VEGF treatment, and administrative documentation across multiple clinical encounters. The evaluated dataset consisted of 24 heterogeneous clinical and administrative documents, including outpatient encounter notes, OCT summaries, operative reports, injection procedure documentation, medication records, billing-related documents, and reimbursement-associated administrative forms. These documents collectively represented a longitudinal real-world administrative workflow involving diagnosis, treatment planning, intravitreal injection, follow-up monitoring, and claim submission.

The unit of analysis for the feasibility evaluation was defined at the level of documents, structured fields, and clinical encounters rather than individual patients. This design choice reflects the technical objective of the study, namely assessing whether the proposed framework could consistently transform unstructured clinical records into structured outputs while satisfying administrative validation constraints across multiple encounters within a realistic workflow setting.

Field-level information extraction performance was evaluated by comparing system-generated structured outputs against expert-curated reference annotations established by retinal specialists and clinical informatics researchers. Agreement was assessed using exact field matching and reported as raw agreement counts and corresponding descriptive percentages. Separate evaluations were performed for clinical entity fields, including best-corrected visual acuity, intraocular pressure, OCT-derived biomarkers, diagnoses, and medication information, as well as for administrative descriptors such as procedure codes, encounter dates, billing attributes, and laterality indicators. These measures are reported as engineering-oriented validation indicators intended to characterize within-case system consistency rather than statistically generalizable extraction accuracy.

Administrative compliance evaluation was performed using the neuro-symbolic reasoning layer. Each processed document was evaluated against predefined ICD-10/CPT coding rules, reimbursement eligibility constraints, laterality consistency rules, documentation completeness requirements, and temporal treatment constraints encoded within the knowledge graph and Logical Tensor Network (LTN) framework. Documents failing initial validation were automatically flagged and subsequently reviewed to identify the violated logical constraints and administrative inconsistencies.

Detected validation issues were manually categorized to characterize the types of administrative inconsistencies identifiable by the proposed neuro-symbolic framework. Representative examples included ambiguous or missing laterality information, incomplete procedural descriptors, missing medication identifiers, documentation omissions, and inconsistencies in retreatment scheduling intervals. These analyses were intended to demonstrate the types of workflow-level administrative issues detectable through symbolic reasoning rather than to estimate error prevalence in broader clinical populations.

Workflow impact was evaluated at the encounter level by measuring clinician effort required for documentation under two conditions: (1) conventional fully manual documentation and (2) clinician review and co-signing of AI-generated drafts. Documentation time was recorded during routine workflow operation and summarized using median values and interquartile ranges (IQRs). Reported workload reductions therefore represent descriptive within-case observations illustrating potential operational efficiency gains in this specific implementation setting.

Because the present study focused on technical feasibility and workflow integration, portions of the evaluated dataset were also used iteratively during development and refinement of the knowledge graph structure, logical constraints, and symbolic validation rules. The Logical Tensor Network was therefore not evaluated as a separately trained predictive model using an independent held-out testing cohort. Instead, the feasibility protocol was designed to assess whether the integrated neuro-symbolic framework could operate consistently and transparently across realistic longitudinal documentation scenarios. Consequently, all reported results should be interpreted as engineering feasibility indicators supporting future large-scale validation studies rather than as evidence of clinical effectiveness, economic generalizability, or population-level deployment performance.

## 3 Results

### 3.1 Feasibility evaluation on longitudinal AMD administrative records

The proposed hybrid Neuro-Symbolic and LLM framework was evaluated using the complete longitudinal administrative and clinical record of a single de-identified AMD patient (Subject 1). The evaluated dataset consisted of 24 heterogeneous unstructured PDF documents collected across a three-month treatment period encompassing initial diagnosis, repeated intravitreal anti-VEGF injections, follow-up visits, OCT imaging assessments, operative documentation, and reimbursement-related administrative workflows. Document types included outpatient consultation notes, operative reports, OCT summaries, medication records, injection procedure documentation, and billing-related administrative forms.

The domain-adapted LLM automatically extracted structured clinical and administrative entities from the unstructured records, including best-corrected visual acuity [BCVA; standardized to Early Treatment Diabetic Retinopathy Study (ETDRS) letters], intraocular pressure (IOP), OCT-derived biomarkers such as central macular thickness (CMT), intraretinal fluid (IRF), and subretinal fluid (SRF), as well as procedural details, medication information, laterality attributes, billing codes, and encounter-level administrative descriptors. Structured outputs were subsequently evaluated against expert-curated reference annotations established by retinal specialists and clinical informatics researchers.

Within this feasibility dataset, agreement with the expert-defined reference standard was observed for 59 of 60 extracted clinical entity fields (descriptive agreement: 98.3%) and 29 of 30 administrative descriptor fields (descriptive agreement: 96.7%). These results represent field-level descriptive agreement observations intended to characterize technical feasibility and workflow consistency within the evaluated longitudinal case rather than statistically generalizable extraction performance estimates.

Importantly, although the study involved only a single patient, the evaluated records spanned multiple heterogeneous document types, repeated treatment encounters, longitudinal disease monitoring events, and diverse administrative validation scenarios. The objective of this feasibility evaluation was therefore not to establish population-level generalizability, but rather to assess whether the proposed neuro-symbolic framework could consistently integrate probabilistic LLM extraction with symbolic administrative reasoning across realistic longitudinal ophthalmic workflows.

All evaluated documents satisfied predefined rule-based validation criteria within the feasibility dataset. Nevertheless, the limited single-case design represents an important limitation of the present study. Future work will require multicase and multicenter validation involving larger and more diverse ophthalmic administrative datasets to evaluate robustness, generalizability, workflow scalability, and deployment performance across broader clinical environments.

Three representative approaches for administrative validation in AI-assisted ophthalmic workflows (Table 4) include conventional regex/rule-based systems, LLM-only extraction approaches, and the proposed Neuro-Symbolic+LLM framework. The comparison focuses on four key operational dimensions relevant to real-world healthcare deployment: explainability, uncertainty handling, rule consistency, and auditability.

**Table 4:** Comparative administrative validation strategies.

Method	Explainability	Handles Uncertainty	Rule Consistency	Auditability
Regex/rule engine	Moderate	No	Moderate	Moderate
LLM-only extraction	Low	Yes	Variable	Low
Proposed Neuro-Symbolic + LLM	High	Yes	High	High

Conventional regex or hard-coded rule engines provide moderate explainability because the validation logic is explicitly predefined and human-readable. However, these systems rely on deterministic binary matching and therefore have limited capability to process ambiguous, incomplete, or heterogeneous clinical text. Their performance may also degrade when documentation styles vary or when administrative workflows become increasingly complex, resulting in only moderate rule consistency and auditability.

LLM-only extraction approaches demonstrate strong capability for handling uncertainty due to their probabilistic natural language understanding abilities. These systems can flexibly interpret heterogeneous clinical narratives and extract structured information from unstructured records. Nevertheless, because they lack an explicit symbolic reasoning or validation layer, their outputs may exhibit variable rule consistency and reduced auditability. In addition, the internal reasoning process of large language models is often insufficiently transparent for high-stakes administrative and reimbursement workflows.

In contrast, the proposed Neuro-Symbolic+LLM framework combines probabilistic language understanding with explicit symbolic reasoning and rule-based validation. This hybrid architecture enables uncertainty-aware extraction while simultaneously enforcing consistent administrative constraints through Logical Tensor Network-based soft logical reasoning. As a result, the proposed framework achieves high explainability and auditability because every validation outcome can be traced to interpretable logical predicates and predefined administrative rules. The integration of symbolic constraints with LLM-derived outputs therefore provides a more reliable and transparent approach for automated ophthalmic administrative workflows, particularly in settings requiring coding consistency, reimbursement validation, and regulatory compliance.

### 3.2 Neuro-Symbolic knowledge representation and logical reasoning

The ophthalmic knowledge graphencodes core clinical and administrative entities involved in age-related macular degeneration care, including diagnoses, procedures, medications, imaging findings, laterality attributes, and reimbursement constraints. Relationships between entities represent clinically and administratively valid associations, such as diagnosis–procedure compatibility, laterality consistency across clinical and billing records, and prerequisite conditions for reimbursement eligibility. This structured representation enables explicit modeling of domain knowledge that is not readily captured by purely statistical models.

Logical constraints were implemented using a Logic Tensor Network framework to enforce deterministic validation over the structured outputs generated by the large language model. The LTN layer operates on KG entities and relations to ensure internal consistency, regulatory compliance, and auditability of administrative decisions. Rather than generating new content, the neuro-symbolic component functions as a rule-based verifier that accepts, rejects, or flags extracted items based on predefined logical conditions.

LTN rules included:

- a treatment procedure is considered valid only if a compatible diagnosis is present prior to the procedure;
- laterality must be consistent across diagnosis, procedure, and billing codes; and
- reimbursement eligibility requires satisfaction of both clinical validity and coding compliance constraints.

To formally express the logical constraints imposed by the neuro-symbolic validation layer, we adopt a pseudo-mathematical notation inspired by Logic Tensor Networks (LTNs). In this representation, the ophthalmic knowledge graph consists of individuals (constants) corresponding to clinical encounters (e), diagnoses (d), procedures (p), and billing codes (b). The full set of core predicates, functions, and logical connectives used in the LTN framework are summarized in Table 5. Logical predicates are interpreted under LTN fuzzy semantics, where each predicate evaluates to a real-valued truth degree in the interval [0, 1], rather than a binary true/false outcome.

**Table 5.** Pseudo-math LTN notation for representative rules.

Notation and Definition
Pseudo-math LTN notation for representative rules
# Let the KG contain individuals (constants) for encourters e, diagnoses d,procedures p,and billing codes b
Predicates are interpreted in [0,1] under LTN fuzzy semantics.
Predicates/functions(examples):
Dx(e,d):diagnosis d is asserted in encounter e
Proc(e,p):procedurepis performed in encounter e
Billed(e,b):billing code b is assigned in encounter e
Compatible(d,p):diagnosis d is clinically compatible with procedurep
Before(d,p):diagnosis time precedes procedure time (or is documented prior).
Lat(x,ℓ):item xhas lateralityℓ∈{L,R,B,U}
ICD10(d,c),CPT(p,c):mapping from clinical items to code c
CodeValid(c):code c is valid under the coding ruleset
Eligible(e):encounter e is reimbursement-eligible
ValidProc(e):procedures in encounter e satisfy clinical prerequisites
CodingOK(e):encounter e satisfies coding constraints
We writeand as LTN quantfiers, andV as fuzzy connectives.

The notation uses a set of domain-specific predicates and functions to encode clinical and administrative knowledge. For example, Dx(e,d) denotes that diagnosis d is asserted in encounter e, while Proc(e,p) indicates that procedurep is performed during encountere. Administrative information is captured through predicates such as Billed(e,b), which represents the assignment of billing codebto encounter e.Clinical compatibility and temporal ordering are modeled using predicates such as Compatible(d,p), indicating that diagnosis d is clinically appropriate for procedure p, and Before(d,p),denoting that the diagnosis is documented prior to the procedure.

Laterality information,which is critical in ophthalmicpractice, is represented by the predicate Lat(a,ℓ), where belongs to the set {L,R,B,U},corresponding to left,right, bilateral, or unspecified laterality. Mappings between clinical concepts and standardized codes are expressed using predicates such as ICD10(d,c) and CPT(p,c), while CodeValid(c) indicates that a given code c conforms to the applicable coding ruleset.

Higher-level predicates are used to summarize complex constraints. Specifically, ValidProc(e) denotes that all procedures within encounter e satisfy required clinical prerequisites, CodingOK(e) indicates that all assigned codes comply with coding standards, and Eligible(e) represents that the encounter meets the criteria for reimbursement.

Logical formulas are expressed using LTN quantifiers(e.g.,universal and existential quantification) and fuzzy logical connectives(e.g.,conjunction and disunction). Under this framework,logical rules act as soft constraints whose degrees of satisfactionaare evaluated continuously,enabling robust and explainable validation of LLM-generated structured.puts in the presence of uncertainty or partial information.

These rules provide transparent and explainable safeguards against logical inconsistencies and coding errors, supporting reliable administrative automation in high-stakes clinical settings. A high-level schematic of the knowledge graph and representative logical rules are provided for illustration, while full KG specifications are maintained outside the main manuscript scope.

### 3.3 Domain adaptation, fine-tuning, and data governance

The domain-adapted DeepSeek-R1 model was fine-tuned using a curated corpus composed exclusively of non–patient-specific materials, including publicly available ophthalmology clinical guidelines, de-identified and template-based clinical documentation examples, administrative coding manuals (ICD-10 and CPT), and synthetic or expert-authored instructional samples. No patient-specific electronic medical records, operative reports, or billing documents were used during model training or fine-tuning. Patient-level clinical and administrative documents were introduced only at inference time through a retrieval-augmented generation (RAG) mechanism, which provided contextual grounding for information extraction without modifying model parameters. All retrieved documents underwent prior de-identification in accordance with institutional protocols, and their use was approved under the study's data governance framework. This separation between model training, domain adaptation, and inference-time retrieval ensured compliance with privacy requirements while enabling accurate and context-aware extraction from real-world administrative workflows.

Representative de-identified prompts and their corresponding structured JSON outputs are provided in Supplementary File S3, together with an illustrative comparison against a non-domain-adapted LLM for qualitative reference. This example illustrates how domain adaptation and neuro-symbolic validation enable deterministic, structured, and audit-ready outputs, in contrast to the loosely formatted and potentially ambiguous responses produced by a non-domain-adapted LLM.

### 3.4 Rule-Guided compliance and error reduction

Structured outputs generated by the LLM were subsequently evaluated by the neuro-symbolic reasoning layer, which applied first-order logical constraints derived from the AMD knowledge graph and reimbursement policies. These rules verified temporal constraints on injection intervals, cross-checked eye laterality and dosage consistency, and assessed documentation completeness required for insurance claims and medico-legal auditing.

Across the 24 documents, the system identified eight minor inconsistencies (3.4% of documents), including ambiguous laterality references and missing medication lot numbers. All identified issues were automatically flagged for clinician review and corrected within the same processing cycle. Following correction, all documents met ICD-10 and CPT compliance requirements during the observation period. These findings demonstrate rule-based feasibility in identifying and resolving administrative inconsistencies in a single-case setting and should not be interpreted as population-level estimates of compliance or denial rates. In accordance with the document-level compliance assessment defined in Section 2.11, the compliance and error-identification results presented in this subsection reflect rule-based feasibility outcomes at the document level, illustrating the system's ability to detect and resolve administrative inconsistencies within this implementation context.

### 3.5 Automated documentation and billing generation

After successful neuro-symbolic validation, the large language model, constrained by symbolic reasoning outputs, generated complete clinical documentation and billing narratives for each encounter, including operative notes, longitudinal comparison summaries, and reimbursement-ready claim descriptions.

Clinician effort was evaluated as the time required to review, verify, and electronically co-sign AI-generated documentation. In this feasibility case, fully manual documentation required a median of 17 min [interquartile range (IQR): 15–20 min] per encounter, whereas review and verification of AI-generated drafts required a median of 2 min (IQR: 1–3 min). This corresponded to an approximate within-case reduction in documentation workload of 88%.

Among the 24 processed documents, all AI-generated claims satisfied ICD-10 and CPT compliance requirements after neuro-symbolic validation and were accepted during initial administrative review. Rule-based consistency checks identified potential documentation inconsistencies in 8 of 24 documents prior to final submission, including missing temporal descriptors and coding conflicts.

These findings represent descriptive observations from a single-patient feasibility study and should not be interpreted as population-level performance estimates. As described in Section 2.11, workflow metrics are reported as encounter-level feasibility indicators intended to demonstrate technical integration and clinical workflow compatibility rather than statistical generalizability.

### 3.6 Economic outcomes and administratively efficientness

Economic implications were explored using a time-driven activity-based costing approach from a provider administrative perspective. Based on local wage assumptions, the observed reduction in documentation time for this case corresponded to an estimated within-case labor saving of approximately 52 CNY (≈7 USD) per encounter.

When extrapolated illustratively to a typical AMD treatment schedule of six to eight intravitreal injections per year, this case would correspond to an estimated 42–56 USD in annual documentation-related labor savings. Additional potential savings from reduced rework and claim resubmission were noted but not formally modeled. All economic values presented here are illustrative, case-based estimates and should not be interpreted as evidence of generalized administratively efficientness.Following the feasibility-oriented economic perspective defined in Section 2.11, all reported cost values represent illustrative, case-specific estimates derived from observed documentation time differences and are not intended to establish generalized administratively efficientness.

Table 6 presents a functional comparison of different architectural configurations used for automated administrative validation in ophthalmic clinical workflows. The comparison evaluates the contribution of individual system components across four critical operational capabilities: structured information extraction, rule validation, explainability, and administrative error detection.

**Table 6:** Functional contribution of framework components.

Configuration	Structured extraction	Rule validation	Explainability	Administrative error detection
LLM only	✓	✗	Limited	Partial
LLM+hard-coded rules	✓	Partial	Moderate	Moderate
Proposed Neuro-Symbolic+LTN	✓	✓	High	High

The LLM-only configuration demonstrated strong capability for structured information extraction from heterogeneous clinical documents due to the language understanding ability of large language models. However, because this configuration lacked an explicit symbolic reasoning layer, it was unable to perform deterministic administrative rule validation. Consequently, explainability remained limited, and only partial administrative error detection could be achieved, particularly for complex workflow inconsistencies such as laterality mismatches or reimbursement eligibility conflicts.

The LLM combined with hard-coded rules improved administrative validation capability by introducing predefined deterministic constraints. This hybrid configuration enabled moderate explainability and improved detection of common administrative inconsistencies. Nevertheless, because conventional rule engines typically rely on binary logic and manually maintained rules, their ability to handle uncertain or ambiguous extraction outputs remained limited. As workflow complexity increased, rule maintenance and consistency management also became more challenging.

In contrast, the proposed Neuro-Symbolic+Logical Tensor Network framework integrated probabilistic LLM-based extraction with differentiable symbolic reasoning and soft logical constraint evaluation. This architecture enabled simultaneous support for structured extraction, uncertainty-aware rule validation, and interpretable administrative reasoning. By combining explicit symbolic predicates with continuous truth-value evaluation, the proposed framework achieved high explainability and high administrative error detection capability while maintaining consistent validation behavior across heterogeneous longitudinal clinical records. These findings suggest that the neuro-symbolic architecture provides a more robust and clinically transparent solution for AI-assisted administrative automation compared with purely neural or purely rule-based approaches.

### 3.7 Clinical workflow and quality impact

Beyond quantitative workflow measures, the system improved administrative transparency by generating logic-verified, longitudinal summaries of BCVA, IOP, and OCT biomarkers at each visit. These summaries facilitated rapid clinician review and simplified quality assurance and regulatory documentation. Across the evaluated structured entity fields, the extraction pipeline achieved a micro-averaged precision of 0.98, recall of 0.97, and F1-score of 0.98 within the feasibility dataset.

No diagnostic or therapeutic decisions were automated. All AI-generated outputs were independently reviewed and electronically co-signed by the attending ophthalmologist, ensuring that clinical judgment remained central to patient care. Collectively, these observations demonstrate the technical feasibility of integrating neuro-symbolic reasoning with LLM-based language processing to support administrative workflows in AMD management (Table 7). In line with the evaluation protocol described in Section 2.11, the workflow and quality observations reported here serve to contextualize the technical feasibility and administrative impact of the system rather than to imply changes in diagnostic or therapeutic decision-making.

**Table 7:** Descriptive within-case feasibility outcomes of the neuro-symbolic+LLM framework.

Dimension	Metric	Baseline (Manual)	AI-Assisted (Case-Specific)	Interpretation
Data extraction	Agreement with expert reference standard (clinical fields)	–	59/60 fields (98.3%)	Descriptive within-case agreement
Data extraction	Agreement with expert reference standard (administrative fields)	–	29/30 fields (96.7%)	Descriptive within-case agreement
Compliance & validation	Documents meeting ICD-10/CPT requirements	–	24/24 documents (100%)	Feasibility observation
Error identification	Documents with flagged inconsistencies	–	8/24 documents (33.3%)	Rule-based detection prior to submission
Documentation time	Median clinician time per encounter	17 min (IQR 15–20)	2 min (IQR 1–3)	Within-case workload reduction
Economic impact	Estimated labor saving per encounter	–	≈52 CNY (≈7 USD)	Illustrative case-based estimate
Workflow quality	Physician review and co-signing	Full manual authorship	Rapid review and co-sign	Maintains clinician accountability

All values represent descriptive, within-case observations from a single-patient feasibility study and should not be interpreted as population-level estimates or statistically generalizable results.

Table 2 summarizes the descriptive, within-case feasibility outcomes observed after deploying the Neuro-Symbolic and Large Language Model (LLM) framework for administrative automation in the management of age-related macular degeneration (AMD). Rather than presenting population-level performance metrics, the table reports case-specific agreement measures, workflow indicators, and illustrative economic estimates derived from a single-patient longitudinal evaluation.

Specifically, the table shows that the system achieved high descriptive agreement with an expert reference standard for both clinical entity fields (e.g., BCVA, IOP, and OCT biomarkers) and administrative descriptors (e.g., procedure codes and billing fields), expressed as raw field counts and percentages. Rule-based validation identified and corrected a small number of documentation inconsistencies, resulting in full compliance with ICD-10 and CPT reimbursement requirements for all documents processed in this case. From a workflow perspective, the table highlights a substantial within-case reduction in clinician documentation workload, measured as a decrease in median time required per encounter when reviewing AI-generated drafts compared with manual documentation. The associated labor cost savings are reported as illustrative, case-based estimates derived from time-driven activity-based costing assumptions. In addition, Table 2 documents qualitative workflow outcomes, including maintained physician oversight through rapid review and electronic co-signing. Collectively, the table provides a concise overview of the system's technical feasibility, administrative impact, and documentation quality in a real-world AMD care setting, without implying statistical generalizability.

### 3.8 Break-Even and threshold analysis

To contextualize the illustrative administrative savings observed in this feasibility study, a break-even threshold analysis was performed to explore the relationship between potential system development costs and the number of patients required to offset those costs through administrative efficiency gains. This analysis does not assume specific development expenditures but instead examines a range of hypothetical cost scenarios to support interpretability and planning.

Based on the within-case observations, documentation-related administrative savings were estimated at approximately 42–56 USD per patient per year, assuming a typical AMD treatment schedule of six to eight intravitreal injections annually. Using these values, the break-even point can be expressed as: Break-even patients per year=Development cost/Annual administrative savings per patient.

Table 8 illustrates break-even thresholds under three representative development cost scenarios (5,000 USD, 20,000 USD, and 50,000 USD), spanning small-scale prototype development to more robust institutional deployment.

**Table 8:** Illustrative break-even thresholds under different development cost scenarios.

Assumed Development Cost (USD)	Annual Savings per Patient (USD)	Break-Even Patients per Year
5,000	42–56	∼90–120
20,000	42–56	∼360–480
50,000	42–56	∼900–1,190

This threshold analysis demonstrates that even when conservative administrative savings are assumed, the number of patients required to offset development costs may be attainable in medium- to large-volume ophthalmology practices or hospital networks. Importantly, these estimates are illustrative and feasibility-oriented, as they do not account for excluded cost components or potential economies of scale. Nonetheless, the analysis provides a transparent framework for interpreting administrative efficiency gains in relation to system development investment and supports informed decision-making for future pilot studies and institutional adoption.

Beyond aggregate agreement metrics, we performed a descriptive analysis of system behavior at the field level to better characterize error patterns. Agreement was analyzed by category, including diagnosis labels, laterality attributes, procedure codes, medication fields, imaging findings, and administrative coding elements. We report field-level agreement distributions across encounters, summarized using ranges and mean ± standard deviation, rather than relying solely on binary pass/fail outcomes. This analysis revealed that laterality-linked fields and diagnosis–procedure code mappings were the most common sources of discrepancy prior to neuro-symbolic validation, whereas core clinical entity identification was comparatively robust. These findings highlight the value of deterministic constraint enforcement in addressing systematic administrative error modes that are not apparent from aggregate accuracy alone.

## 4 Discussions

This study provides a technical feasibility demonstration that artificial intelligence can be applied to automate healthcare administrative workflows in ophthalmology, rather than to influence diagnosis or treatment selection. Within the context of a single-case, longitudinal evaluation, the proposed Neuro-Symbolic and LLM framework demonstrated the ability to improve documentation completeness, coding correctness, reimbursement compliance, and administrative efficiency in the management of AMD. These outcomes directly address the administrative burden surrounding clinical care, which represents a substantial but often underrecognized source of inefficiency in chronic disease management. Clinician review time may exhibit learning effects, it may decrease with familiarity or increase if trust degrades after encountering errors. Given the single-case feasibility design, we report observed review times descriptively and do not draw inferential conclusions. Future multi-clinician studies should incorporate a wash-in period and repeated-measures analysis to quantify learning and trust dynamics.

As a technical feasibility study, the primary objective was to evaluate system operability, auditability, and cost plausibility under real-world administrative workflows, rather than to estimate population-level effects. A single-subject longitudinal design was therefore selected to enable fine-grained tracing of document processing, validation logic, and timing measurements. The break-even point was calculated under the assumption that the system development cost represents a one-time investment amortized over the expected deployment lifetime of the system. Under a multi-year deployment scenario, the effective denominator increases proportionally with the number of years of operation, thereby substantially reducing the per-patient break-even threshold. Consequently, the estimated break-even point is sensitive to the assumed deployment horizon and should be interpreted as illustrative rather than definitive, reflecting a plausible economic scenario rather than a fixed cost-effectiveness guarantee.

The primary contribution of this work lies in demonstrating within-case reductions in documentation workload alongside improvements in documentation quality. By decreasing the clinician time required to review and finalize administrative records, the system supports administrative workflows surrounding clinical care and reduces repetitive clerical tasks. Importantly, this study does not evaluate diagnostic accuracy or treatment decision-making, nor does the system automate clinical reasoning. All diagnostic and therapeutic decisions remained fully under physician control. The framework operates downstream of clinical judgment, transforming unstructured records into structured, logic-verified documentation that remains subject to clinician review and electronic co-signing. Accordingly, any observed time savings or economic implications should be interpreted strictly as consequences of improved administrative efficiency, rather than as changes in clinical practice or patient outcomes.

The feasibility evaluation yields several insights relevant to AI-enabled administrative automation in chronic disease management. First, observed error modes were predominantly administrative rather than clinical, with the most consequential failures arising from laterality mismatches and coding constraint violations rather than missed clinical entities, underscoring the importance of deterministic neuro-symbolic validation. Second, auditability emerged as a key adoption lever: physician acceptance was strongly influenced by the availability of traceable, field-level justifications and efficient review-and-cosign workflows, rather than by raw automation speed alone. Third, the longitudinal and repetitive nature of chronic disease care—such as scheduled intravitreal anti-VEGF treatments in age-related macular degeneration—produces recurring documentation patterns, making these settings particularly well suited for structured administrative automation with high potential return on investment.

Beyond workflow efficiency, the integration of symbolic reasoning with LLM-based language processing enhanced coding accuracy and reimbursement compliance through explicit, rule-based validation. The system's ability to identify and flag documentation inconsistencies prior to claim submission has practical implications for reducing administrative rework and minimizing reimbursement delays. In the context of increasingly complex and value-based reimbursement models, such improvements in documentation integrity and auditability may help ensure that clinically appropriate services are accurately represented and reimbursed without increasing administrative burden for providers.

## 5 Limitations and future research

Despite demonstrating promising technical feasibility and workflow integration, several important limitations of the present study should be acknowledged. First, this investigation was designed as an engineering-oriented, single-case feasibility study involving one de-identified patient with age-related macular degeneration and 24 heterogeneous longitudinal clinical and administrative documents collected within a single healthcare system. Although the dataset provided temporally rich and operationally realistic workflow scenarios, the limited sample size and single-center design inherently restrict the generalizability of the findings. Consequently, the reported results should be interpreted as descriptive feasibility observations rather than statistically generalizable estimates of clinical or administrative performance.

Second, portions of the evaluated dataset were iteratively involved in the refinement of the knowledge graph structure, symbolic validation rules, and Logical Tensor Network constraints. While this iterative engineering process was appropriate for framework development and feasibility validation, it may introduce workflow-specific optimization bias. Independent multicenter evaluations using larger and more diverse ophthalmic datasets will therefore be necessary to assess robustness, scalability, and external validity across institutions, documentation styles, reimbursement systems, and patient populations.

Third, although the proposed neuro-symbolic framework integrates explainable symbolic reasoning with domain-adapted large language model extraction, system performance remains dependent on the quality and completeness of source documentation. Ambiguities, inconsistencies, or omissions within clinical notes, imaging summaries, or administrative records may propagate through downstream processing despite symbolic validation. Continued refinement of uncertainty quantification strategies, data quality control mechanisms, and human-in-the-loop verification workflows will therefore remain important for safe real-world deployment.

Fourth, the economic evaluation focused primarily on direct reductions in clinician documentation workload and associated administrative labor costs. Broader implementation-related considerations—including infrastructure requirements, deployment costs, maintenance expenses, workflow integration overhead, and long-term operational sustainability—were not comprehensively evaluated in the present study. Future investigations should adopt a more comprehensive health-economic framework incorporating longitudinal operational costs, organizational implementation factors, and cost–utility metrics such as cost per quality-adjusted life year (QALY).

Finally, the proposed framework was evaluated within the regulatory, coding, and reimbursement environment of a single healthcare system in China. Adaptation to other jurisdictions will likely require customization of knowledge graph structures, administrative rule sets, coding ontologies, compliance mechanisms, and payer-specific validation policies. Collaboration with regulatory agencies, insurers, and international standards organizations will therefore be essential to support broader clinical translation and interoperability.

Future work will focus on multicenter validation involving larger and more heterogeneous ophthalmic populations, prospective real-world workflow evaluation, and integration of additional ophthalmic disease workflows beyond AMD. Comparative studies evaluating neuro-symbolic architectures against LLM-only pipelines and conventional rule-based systems will also be important for quantifying the relative contributions of explainability, uncertainty handling, and administrative validation performance. In addition, incorporation of multimodal ophthalmic data sources—including fundus photography, genetic information, and lifestyle-related risk factors—may further enhance predictive capability and administrative precision. Collectively, these future directions may support the development of scalable, transparent, and clinically deployable neuro-symbolic administrative AI systems for ophthalmology and other chronic disease domains.

In sum, while this single-case study provides a compelling proof of concept, realizing the full potential of the proposed framework will require broader clinical validation, comprehensive economic assessment, and proactive regulatory engagement, ensuring that the technology remains effective, equitable, and trustworthy as it is scaled across diverse healthcare settings.

## 6 Conclusion

This study presents a single-case technical feasibility evaluation of a hybrid Neuro-Symbolic and Large Language Model framework designed to automate healthcare administrative workflows in the management of age-related macular degeneration. By focusing explicitly on documentation processing, coding validation, and reimbursement-related tasks, the proposed system demonstrates how artificial intelligence can be applied to reduce administrative burden without intervening in diagnostic reasoning or treatment decision-making.

Within the evaluated case, the framework reliably transformed heterogeneous, unstructured clinical documents into structured, logic-verified administrative outputs, supporting documentation completeness, coding correctness, and reimbursement compliance. The integration of symbolic reasoning with LLM-based language processing enabled transparent rule-based validation, allowing administrative inconsistencies to be identified and corrected prior to claim submission. Observed reductions in clinician documentation workload and illustrative administrative cost offsets highlight the potential operational value of such systems when applied to real-world ophthalmic workflows, while remaining strictly descriptive and context-specific.

Importantly, all findings reported in this study should be interpreted as within-case feasibility indicators rather than generalizable performance or economic effectiveness estimates. The system was evaluated from a provider administrative perspective, and the reported economic metrics are illustrative, excluding development, deployment, and maintenance costs. No claims are made regarding return on investment, clinical outcomes, or population-level cost-effectiveness.

Overall, this work demonstrates that a neuro-symbolic+LLM architecture offers a transparent, auditable, and policy-aligned approach to administrative automation in chronic ophthalmic care. While limited by its single-case design, the study provides a reproducible foundation for future multi-center investigations, controlled comparisons with alternative automation strategies, and broader evaluations across institutional and regulatory settings. As healthcare systems increasingly seek to address administrative inefficiencies, such feasibility-oriented frameworks may play a critical role in enabling scalable, accountable, and responsible AI deployment in clinical administration.

## Data Availability

The original contributions presented in the study are included in the article/Supplementary Material, further inquiries can be directed to the corresponding author.

## Glossary

The following abbreviations are used in this manuscript:

**Table:**

Abbreviation	Full Term	Context/Meaning in the Study
AI	Artificial Intelligence	Computational methods that mimic human cognitive functions; here used to automate clinical documentation, imaging comparison, and insurance claim preparation.
ML	Machine Learning	Subfield of AI enabling systems to learn patterns from data; supports automated extraction and prediction tasks.
LLM	Large Language Model	Advanced neural network trained on extensive text corpora; applied for natural-language understanding, structured data extraction, and automated report generation.
Neuro-Symbolic AI	Neuro-Symbolic Artificial Intelligence	AI paradigm integrating symbolic logical reasoning with neural network pattern recognition, ensuring explainability and rule-based compliance.
AMD	Age-Related Macular Degeneration	Leading cause of irreversible blindness in older adults and the clinical focus of this study.
anti-VEGF	Anti–Vascular Endothelial Growth Factor	Intravitreal injection therapy used to treat neovascular AMD and associated macular edema.
OCT	Optical Coherence Tomography	High-resolution retinal imaging modality for monitoring macular thickness and fluid status.
BCVA	Best-Corrected Visual Acuity	Standardized measure of visual function, reported in ETDRS letters.
IOP	Intraocular Pressure	Key ophthalmic parameter used to monitor ocular health and treatment effects.
ETDRS	Early Treatment Diabetic Retinopathy Study	International standard for visual acuity measurement, used to quantify BCVA.
ICD-10	International Classification of Diseases, Tenth Revision	Global diagnostic coding standard required for insurance reimbursement and health statistics.
CPT	Current Procedural Terminology	Coding system for medical, surgical, and diagnostic services used in billing and claims.
KG	Knowledge Graph	Structured representation of clinical entities, imaging biomarkers, treatment events, and administrative rules enabling neuro-symbolic reasoning.
LTN	Logic Tensor Network	Formalism that encodes first-order logical constraints in differentiable form, used for rule-based validation of clinical and billing data.
TDABC	Time-Driven Activity-Based Costing	Economic evaluation method linking time requirements to actual cost for precise administratively efficientness assessment.
OCR	Optical Character Recognition	Technique to convert scanned or image-based documents (PDFs) into machine-readable text.
